# Cure Behavior and Thermomechanical Properties of Phthalonitrile–Polyhedral Oligomeric Silsesquioxane Copolymers

**DOI:** 10.3390/polym9080334

**Published:** 2017-08-03

**Authors:** Xiaodan Li, Baifeng Yu, Dongxing Zhang, Jing Lei, Zhu Nan

**Affiliations:** 1 School of Materials Science and Engineering, Harbin Institute of Technology, Harbin 150001, China; lixiaodanlixiaodan@126.com; 2 Harbin FRP Institute, Harbin 150029, China; yubaifeng888@163.com (B.Y.); jinglyhby@163.com (J.L.); zhunan0451@126.com (Z.N.)

**Keywords:** phthalonitrile polymers, POSS, thermal stability, char yield, mechanical performance

## Abstract

Phthalonitrile–polyhedral oligomeric silsesquioxane (POSS) copolymers were prepared by adding two different POSS cage mixtures: epoxycyclohexyl POSS (EP0408) and *N*-phenylaminopropyl POSS (AM0281). The cure behavior and properties of these polymers were analyzed and compared using differential scanning calorimetry (DSC), thermogravimetric analysis (TGA), dynamic mechanical analysis (DMA), Fourier transform far infrared (FTIR) measurements, and rheometric studies. The POSS-containing polymers showed higher chemical reactivity, better thermal stability and better mechanical performance in comparison to their unmodified counterparts. All the polymers showed water absorption below 1.5%. As revealed by FTIR measurements, the polymerization products contained triazine ring structures that were responsible for the superior thermal properties exhibited by these POSS-containing polymers.

## 1. Introduction

Fiber-reinforced composites have been widely used over the past several decades in numerous structural applications (e.g., aircraft, missile, ship, and vehicle construction) owing to their good mechanical performance and light weight. These materials have developed quickly due to their potential for future applications. Phthalonitrile polymers [1,2,3,4,5], a new class of thermal materials combining low flammability and high strength, have shown great potential in the aerospace sector as components for maintaining airframe loads in the next generation of aeronautical and space vehicle systems. In comparison to other high temperature-resistant materials, phthalonitrile-based composites [6,7,8] have numerous advantages. These include superior mechanical, thermal, and oxidative stability properties compared to most state-of-the-art thermal composites (e.g., polyimide and phenolic triazine). Additionally, phthalonitrile-based composites do not release any by-products during the curing process and the corresponding prepolymer (B-staged resin) can be prepared and stored with an unlimited shelf life under ambient conditions. Regarding fire resistance properties, phthalonitrile-based composites are among the few materials meeting the U.S. Navy’s stringent requirements articulated in the MIL-STD-2031 directive for the usage of polymer composites aboard Navy submarines.

**Figure 1:**
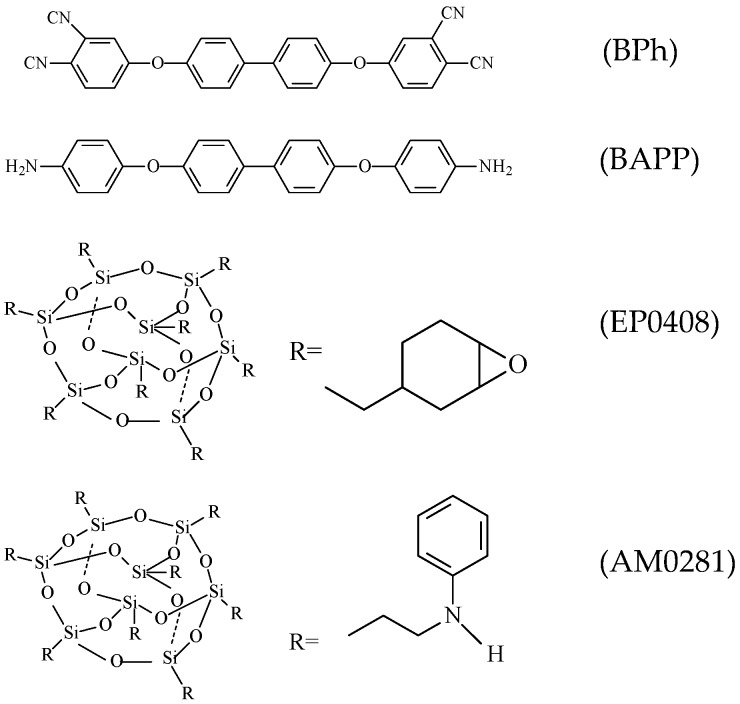
Structures of the phthalonitrile monomer, curing agent, and the two types of phthalonitrile–polyhedral oligomeric silsesquioxane (POSS) used herein.

The phthalonitrile monomer 4,4′-bis(3,4-dicyanophenoxy)biphenyl (BPh) was first synthetized by Keller [1,2] and, since then, extensive work has been carried out by his group and others [9,10,11,12,13,14,15,16,17,18,19,20,21] involving various types of phthalonitrile monomers and curing agents. Thus, new monomers with lower melting temperature were prepared, thereby widening the processing window. However, high temperatures and long curing times are still required to form cross-linked structures during polymerization. Phthalonitrile–polyhedral oligomeric silsesquioxane (POSS) compounds provide unique opportunities to create revolutionary material combinations through a melding of the desirable properties of ceramics and polymers at the 1 nm length scale. These new combinations enable the circumvention of classic material performance trade-offs by exploiting the synergy and properties of materials that only occur on the nanoscale. POSS reagents consisting of an inorganic silsesquioxane cage have multiple reactive groups able to interact with the cyanate group at high temperatures, thereby offering a unique opportunity to prepare nanocomposites with a truly molecular dispersion of inorganic fillers. Additionally, POSS copolymers with enhanced characteristics (e.g., higher glass transition (*T_g_*) temperatures and superior mechanical and oxidative or fire resistant properties) have been prepared in a large range of thermoplastic (e.g., polyethylene, polypropylene, and polycarbonate) and thermosetting (e.g., polyimide, epoxy resins, polyurethane, and cyanate ester resin, among others) polymers [22,23,24,25,26,27,28,29,30]. Kaliavaradhan [31] synthesized tri-phthalonitrile phenyl POSS polymers with high thermal and retardant properties. Bu synthetized POSS-polysulfonamide (PSA, silicon-containing arylacetylene) resins with higher flexural strength and impact fractured energy, by 80.5% and 92.8% respectively [32]. Pen prepared POSS-TiO_2_-epoxy nanocomposites with enhanced thermal stability and UV resistance [33]. Despite these works, phthalonitrile-POSS copolymers have been scarcely studied in literature. Therefore, this work aims to study the effect of POSS on the chemical reactivity and thermal stability properties of phthalonitrile-POSS copolymers. EP0408 and AM0281 are hybrid molecules, each containing an inorganic silsesquioxane core and either eight epoxycyclohexyl or *N*-phenylaminopropyl organic groups at the corners of the cage, respectively. Epoxycyclohexyl POSS (EP0408) and *N*-phenylaminopropyl POSS (AM0281) were chosen with the clear objective of studying the effect of POSS on the cure behavior, thermomechanical properties, and the reactive mechanism of phthalonitrile copolymers. The structures of the monomer and the two POSS reagents used herein are shown in Figure 1.

Aromatic amine was used with EP0408 and AM0281 to co-cure the phthalonitrile monomer such that the main structure of the phthalonitrile polymers is retained. 

## 2. Experimental

### 2.1. Materials

BPh (solid, 99%, without any further purification) was synthesized at the Institute of Chemistry of the Chinese Academy of Sciences. 4,4′-bis(4-aminophenoxy)biphenyl (BAPP, solid, without any further purification) was purchased from Bailingwei Inc. (J&K Scientific Ltd., Beijing, China). The two types of POSS denoted as epoxycyclohexyl POSS (EP0408, semi-solid, without any further purification) and *N*-phenylaminopropyl POSS (AM0281, viscous liquid, without any further purification) cage mixtures were obtained from Hybrid Plastics, Inc. (Hybrid Plastics, Fountain Valley, CA, USA).

### 2.2. Preparation of the Prepolymers, Polymers, and Their Nanocomposites

A mixture of BPh and BAPP (curing agent, 2 wt %) was melted at 260 °C. EP0408 and AM2081 were separately added at varying compositions (0.1, 0.5, 1, 5, and 10 wt %) and the resulting mixtures were stirred for 10 min and cooled down to room temperature to prepare the prepolymers, respectively named the neat prepolymer, EP0408-X prepolymer, and AM2081-X prepolymer, where X indicates the POSS content in wt %. The prepolymers were pulverized before performing differential scanning calorimetry (DSC), dynamic mechanical analysis (DMA) and rheological tests. Neat polymers and those containing EP0408 and AM2081 at 0.1, 0.5, 1, 5, and 10 wt % were cured at 260 (4 h), 300 (8 h), and 325 °C (8 h). Before the DMA experiments, phthalonitrile casting samples were cured in molds (50 × 10 × 2 mm) with an air-circulating oven at 260 (8 h), 300 (8 h), and 325 °C (8 h). The samples were subsequently post-cured under an inert atmosphere of nitrogen at 350 °C (4 h), 350 and 375 °C (4 h), 350 (8 h), and 375 °C (4 h). The rheological behavior of the neat prepolymers and the prepolymer–POSS blends was studied at 280 °C to obtain the complex viscosity–time plots. The neat prepolymers, polymers, and POSS polymers containing EP0408 and AM0281 were studied by Fourier transform far infrared (FTIR) analysis. 

### 2.3. Characterization

DSC experiments were conducted in a flowing nitrogen atmosphere on mixtures containing the monomer and either EP0408 or AM2081 (0.1, 0.5, 1, 5, and 10 wt %). The experiments were conducted within a Perkin–Elmer Pyris-6 DSC calorimeter (Perkin–Elmer, Richmond, CA, USA) at a heating rate of 10 °C/min. DSC curves at different heating rates (5 °C/min, 10 °C/min, 15 °C/min, 20 °C/min) were measured. The activation energies were calculated using the following equation:

(1) $$\frac{d[In(\beta /T_{p}^{2})]}{d[1/T_{p}]}=-\frac{E}{R}$$

where *β* is the heating rate, *T_p_* is the peak temperature of each DSC curve at different heating rates, and *R* is the universal gas constant. Thus, the *E* value was obtained through the linear dependence of *In*(*β*/*T_p_*^2^) on 1/*T_p_*, at various heating rates.

Thermal analysis was performed on polymers with different contents of EP0408 and AM2081, using a TA Instruments SDTQ600 thermogravimetric analyzer (TA Instruments, Eden Prairie, MN, USA). The TGA tests were carried out under flowing nitrogen (100 mL/min) at a scan rate of 10 °C/min. The dynamic storage modulus (*G*’) and damping factor (tanδ) of rectangular phthalonitrile polymer specimens (50 × 10 × 3 mm) were obtained by DMA on a DMS-6100 instrument (NSK Ltd., Tokyo, Japan) with a flowing nitrogen atmosphere and a temperature range of 30–400 °C (4 °C/min, frequency: 10 Hz). Thus, *T_g_* was estimated from the modulus–temperature plots obtained by the DMA. Dynamic viscosity measurements were performed on a TA Instruments AR-2000 rheometer (TA Instruments, Eden Prairie, MN, USA). The water uptakes of the neat polymers and the phthalonitrile–POSS copolymers were monitored under ambient conditions. The FTIR studies were performed on a Nicolet Avatar 370 FT-IR spectrometer (Thermo Fisher Scientific, Grand island, NY, USA) with potassium bromide pellets containing a low amount of sample. 

## 3. Results and Discussion

**Figure 2:**
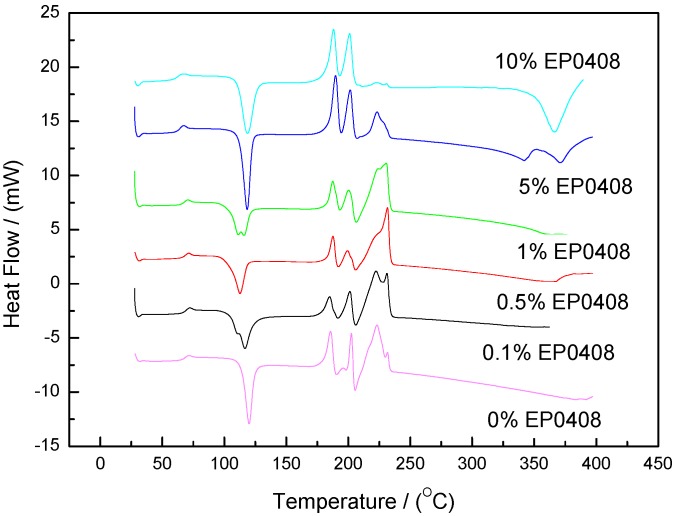
Differential scanning calorimetry (DSC) scans of phthalonitrile monomer (2 wt % BAPP mixtures with 0, 0.1, 0.5, 1, 5, and 10 wt % of EP0408 added at 260 °C).

**Figure 3:**
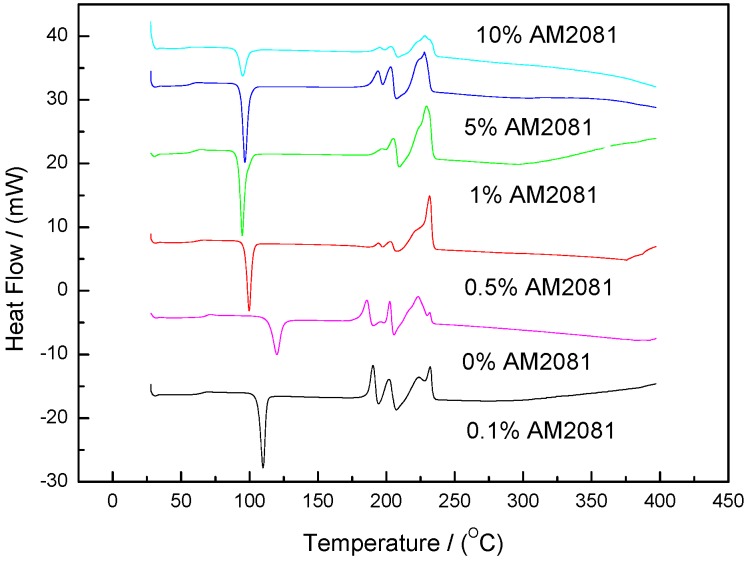
DSC scans of phthalonitrile monomer (2 wt % BAPP mixtures with 0, 0.1, 0.5, 1, 5, and 10 wt % of AM2081 added at 260 °C).

The prepolymers containing EP0408 or AM2081 (0.1, 0.5, 1, 5, and 10 wt %) were studied by DSC, and the results are shown in Figure 2 and Figure 3, respectively. All the DSC scans showed one exothermal peak at 98–127 °C corresponding to the initial reaction between amine and phthalonitrile. The endothermal peaks at 170–250 °C observed were ascribed to the melting of the curing agent, monomer, and prepolymers. Consequently, the position and shape of these endothermal peaks are expected to change with the POSS content. As shown in Figure 2, the area of the exothermal peak at ca. 350–380 °C, the peak ascribed to the formation of networks, increased with EP040 content. However, when using AM2081 (Figure 3), no noticeable exothermal peaks were observed, thereby revealing that cross-linking polymerization is taking place at a very low rate. Considering the above results, it can be determined that the reaction process involves two steps: prepolymerization, since large amounts of prepolymers were produced during the initial reaction, and cross-linking, where cyanogen groups react and form triazine ring structures. The position of the peaks was dependent on the EP0408 or AM0281 contents. The activation energies (peaks at 115–120 °C) for the neat and the EP0408- and AM0281-containing polymers were 135, 129, and 778 kJ/mol, respectively. Thus, the AM0281-containing polymers react more easily at low temperatures compared to the other polymers. The DSC scans of the EP0408-containing polymers showed noticeable peaks at high temperatures (350–370 °C) (Figure 2) with a corresponding activation energy of 1934 kJ/mol. This activation energy was well above that obtained at low temperature, thereby indicating that the reaction at a high temperature is more hindered than that at a low temperature.

**Figure 4:**
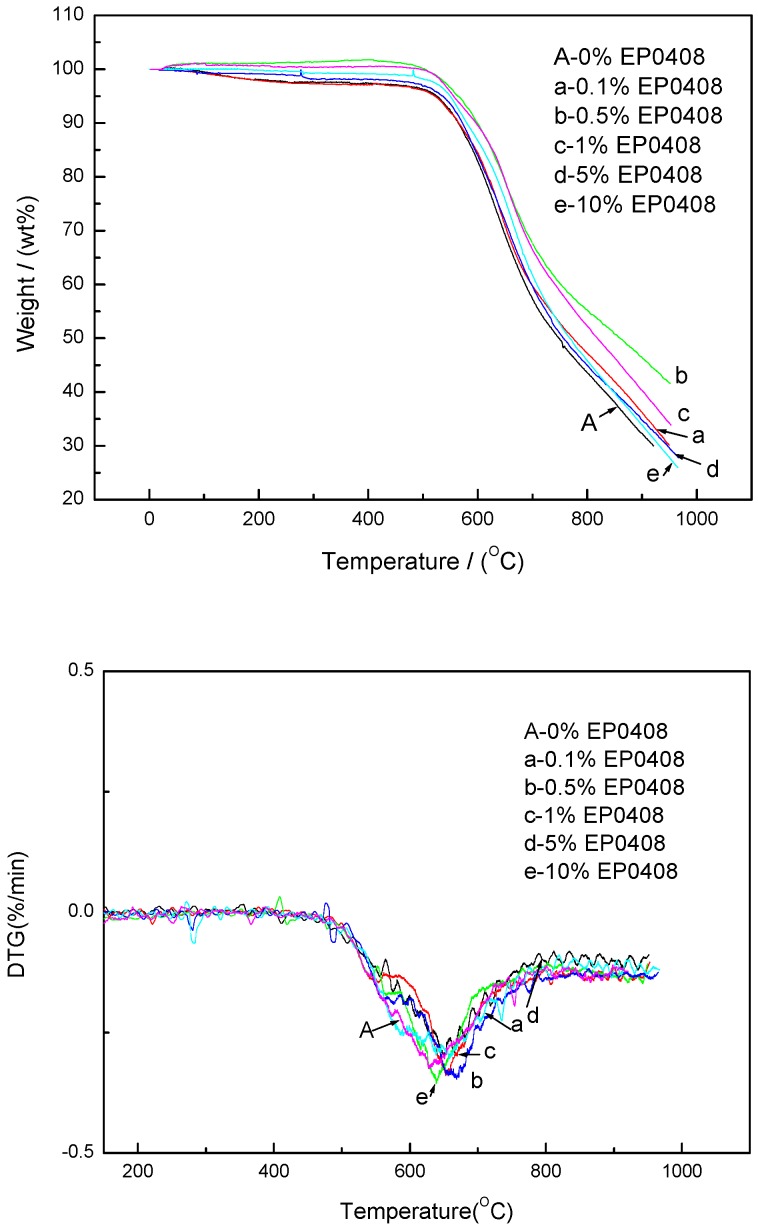
Thermogravimetric analysis (TGA) and Derivative Thermogravimetry (DTG) plots of the neat polymer and the polymers with different EP0408 contents under an air atmosphere.

**Figure 5:**
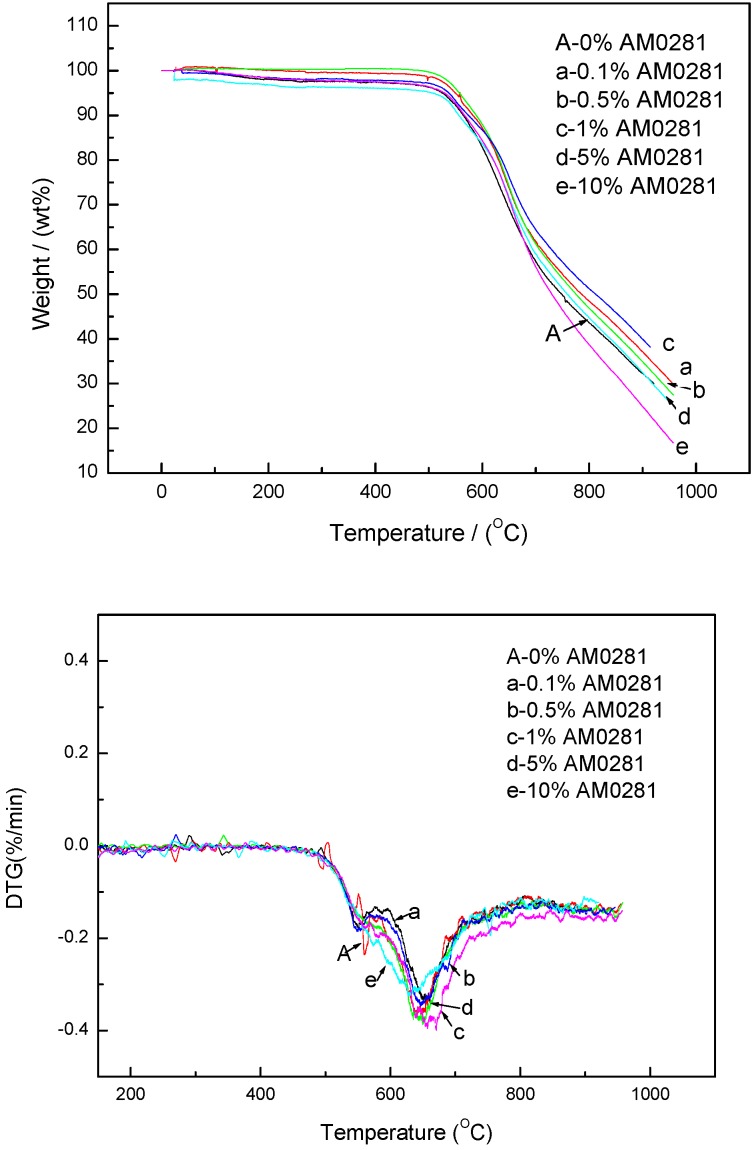
TGA and DTG plots of the neat polymer and the polymers with different AM0281 contents under an air atmosphere.

The TGA profiles of the EP0408- and AM0281-containing materials (0, 0.1, 0.5, 1, 5, and 10 wt %) are shown in Figure 4 and Figure 5, respectively. The EP0408-containing polymers, particularly the EP0408-0.5 polymer, showed higher thermal stability compared to the other polymers. The weight retain percent at 900 °C is about 48% higher than that of the neat polymer (31%). The Derivative Thermogravimetry (DTG) curves exhibit one obvious peak at 640–680 °C. The rate of decomposition became higher from 550 °C and reaches a maximum value at about 640–680 °C.

Most of the AM0281-containing polymers, particularly the AM0281-1 polymer, showed excellent thermal stability compared to the other polymers. The weight retain percent at 900 °C is about 40% higher than that of the neat polymer (31%). However, the AM2081-10 polymer showed poorer thermal stability than the neat polymer. Since AM0281 bears a hindered amine with low reactivity, an excess of this POSS agent in the mixture caused the unreacted amine to decompose at high temperatures, thereby decreasing the stability of the polymer. DTG curves shown in Figure 5 exhibited two obvious peaks at 540–580 °C and 640–680 °C, respectively. These were the temperatures at which the polymers decomposed at the fastest rate.

**Figure 6:**
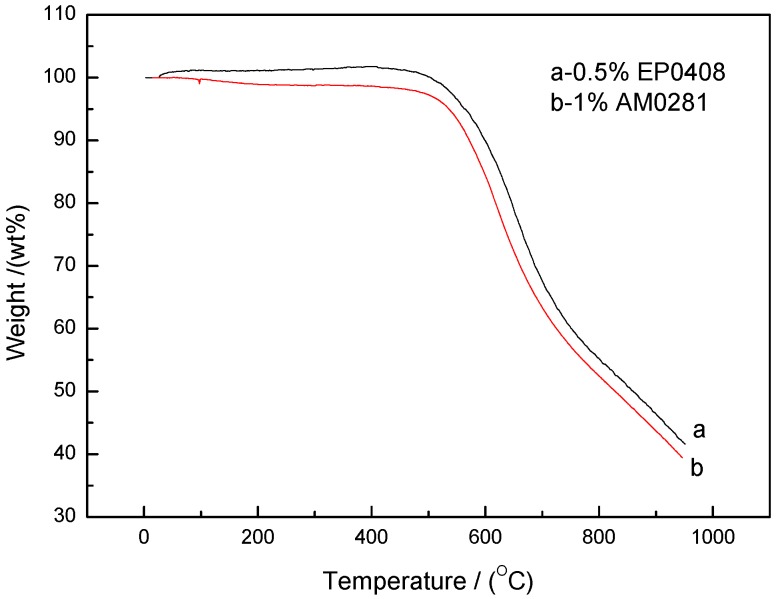
Comparison of the thermal stabilities of the EP0408- and AM0281-containing polymers.

As shown in Figure 4 and Figure 5, the EP0408-0.5 and AM0281-1 samples were the most thermally stable polymers of their respective groups. Both materials were compared in terms of thermal stability in Figure 6 and no significant differences were found between both materials, although the EP0408-containing material showed a slightly higher thermal stability compared to the AM0281-containing polymer.

**Figure 7:**
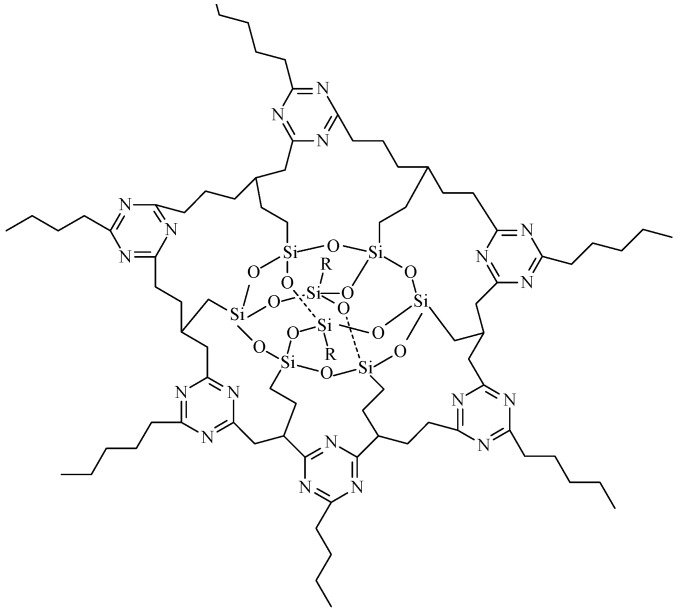
Possible structure of POSS in the triazine ring network.

From the TGA curves of each system, a gradual increase in the content of POSS has the thermal stability increasing at first, before decreasing. When a small amount of POSS agent was added to the system, the POSS ring will enter the network of triazine and make the cross-linked density higher as shown in Figure 7. Thus, the thermal stability is also improved. However, with an excessive amount of POSS agent in the system, the network of the polymer will be highly-branched at the branching point of the POSS ring, the steric hindrance makes triazine difficult to form, and the cross-linked density is decreased. This causes decreased thermal stability.

**Figure 8:**
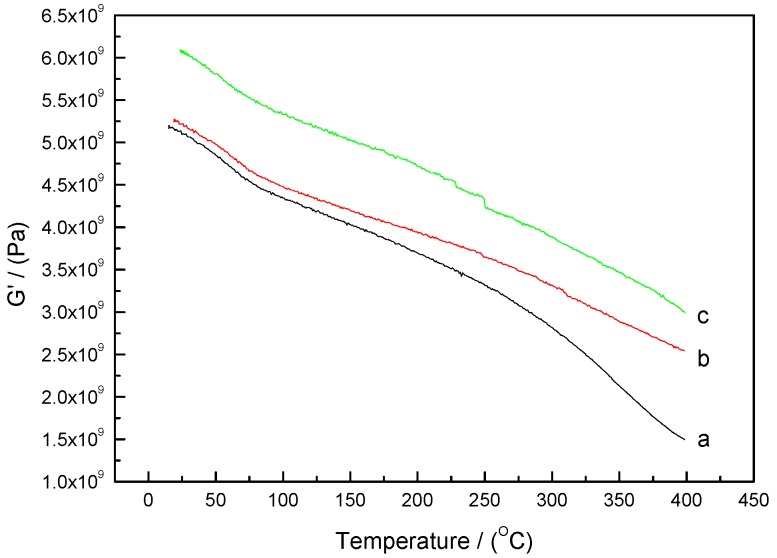
Dynamic storage modulus (*G*’) as a function of temperature for the neat polymer after a post-cure treatment at: (a) 350 °C for 4 h, (b) both 350 and 375 °C for 4 h, and (c) 350 °C for 8 h and 375 °C for 4 h.

**Figure 9:**
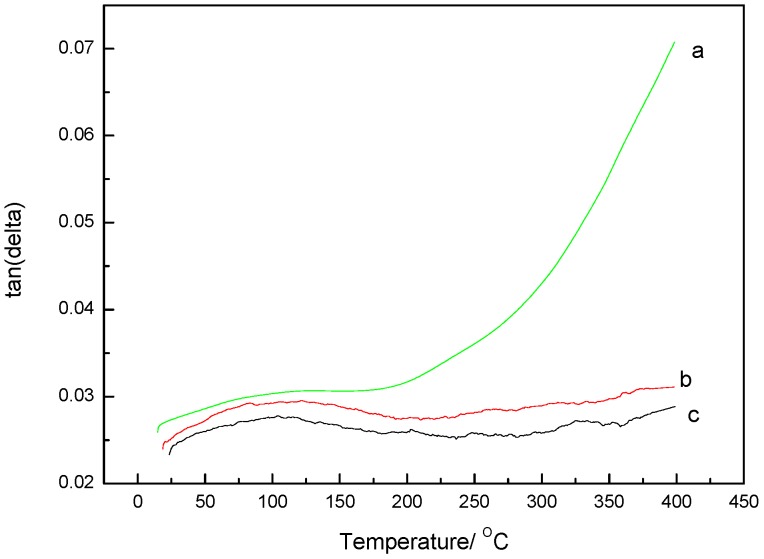
Damping factor (tanδ) as a function of temperature for the neat polymer after post-cure at: (a) 350 °C for 4 h, (b) both 350 and 375 °C for 4 h, and (c) 350 °C for 8 h and 375 °C for 4 h.

DMA measurements were carried out to study the dynamic mechanical properties of the polymers as a function of different post-cured treatments. These preliminary studies were used to identify the curing conditions required to obtain polymers with optimum mechanical properties. Thus, the samples post-cured at 350 °C exhibited a sharp drop in the storage modulus and a peak in the tanδ curves as the temperature increased (curve a in Figure 8 and Figure 9, respectively). This behavior indicates that the polymer shifted from a glassy state to a rubbery state as the segmental motion of the polymer chains increased. While the storage modulus and the tanδ plots were relatively flat for the samples heated at elevated temperatures for long times (curves b and c), the curve c, in particular, showed a higher storage modulus, thereby revealing the presence of a stable cross-linked network hindering the segmental motion of the polymers. This enhancement in the dynamic mechanical properties of the phthalonitrile polymer was attributed to advances in the cross-linking of the thermoset upon elevated temperatures for long times.

**Figure 10:**
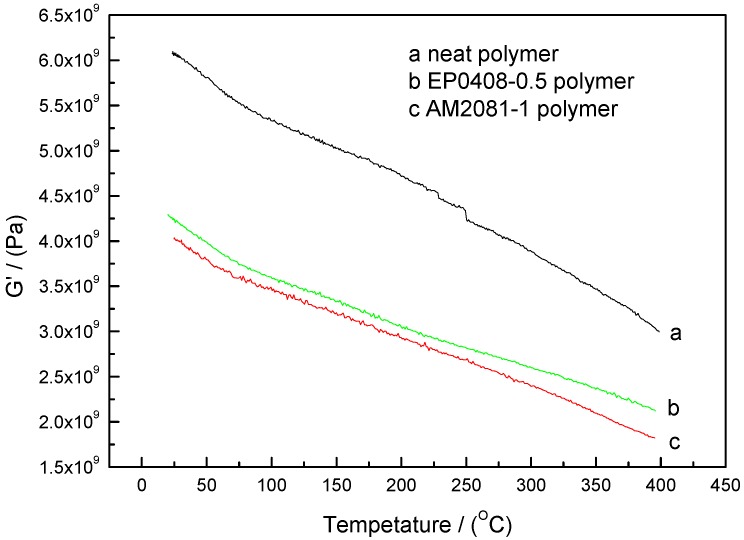
Dynamic storage modulus (*G*’) as a function of temperature for the neat polymer and the polymers containing AM0281 and EP0408 cured at 260 °C for 4 h, 300 °C for 8 h, and 325 °C for 8 h, and subsequently post-cured under an inert atmosphere of nitrogen at 350 °C for 8 h and 375 °C for 4 h. (a) Neat polymer, (b) EP0408-0.5 polymer, and (c) AM0281-1 polymer.

**Figure 11:**
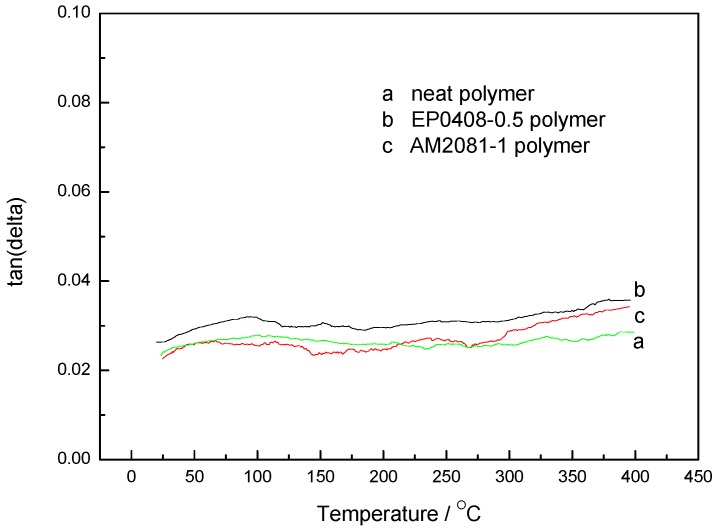
Damping factor (tanδ) as a function of temperature for the neat polymer and the polymers containing AM0281 and EP0408 cured at 260 °C for 8 h, 300 °C for 8 h, and 325 °C for 8 h, and subsequently post-cured under an inert atmosphere of nitrogen at 350 °C for 8 h and 375 °C for 4 h. (a) Neat polymer, (b) EP0408-0.5 polymer, and (c) AM0281-1 polymer.

DMA was carried out on the neat and the AM0281- and EP0408-containing polymers after cure, then post-cured under treatments to evaluate *T_g_* of the cured polymers. As shown in Figure 10, in all cases, the dynamic storage modulus gradually decreased with temperature, which was attributed to a stress relaxation of the polymer network. In comparison with the neat polymer, the storage moduli of the polymers containing EP0408 and AM0281 decreased at significantly lower rates, thereby indicating that temperature had less influence on these polymers. However, the POSS-based polymers, particularly those containing AM0281, showed a lower modulus. Figure 11 shows the damping factor plots and that all the polymers showed a similar *T_g_*, thereby revealing that, in all cases, a stable cross-linked network was formed, hindering the segmental motion.

**Figure 12:**
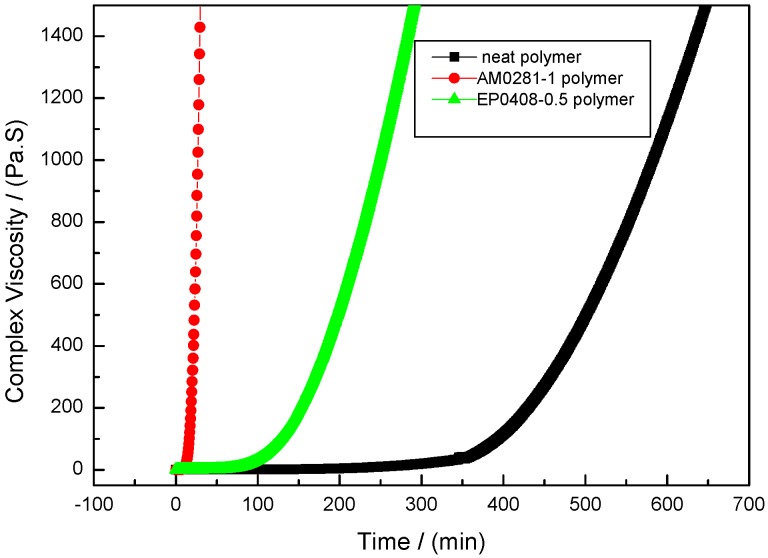
Complex viscosity at 280 °C as a function of time for the neat polymer, the EP0408-0.5 polymer, and AM0281-1 polymer.

The complex viscosity changes accompanying the phthalonitrile polymerization reaction were investigated by performing isothermal rheometric measurements at 280 °C on the neat and phthalonitrile–POSS copolymers (Figure 12). The phthalonitrile–POSS copolymers, especially the AM0281-containing materials, showed a more rapid increase in their viscosity compared to the other polymers. The phthalonitrile–POSS copolymers cured in a far shorter time than that of the neat polymer. As expected, the phthalonitrile–POSS copolymers showed high reactivity and cured at a high rate while maintaining their thermal stability.

**Figure 13:**
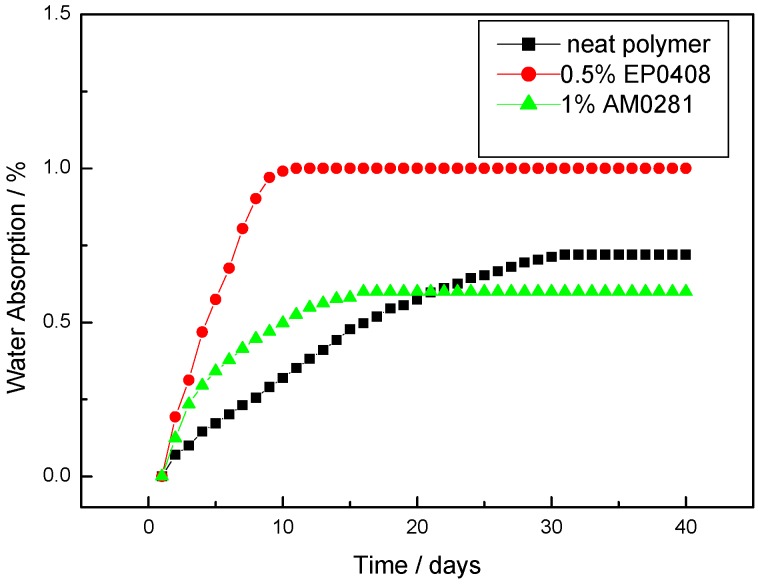
Water absorption of the phthalonitrile polymer and the phthalonitrile–POSS copolymers in ambient conditions.

The water absorption under ambient conditions of the three polymers cured at 260 (8 h), 300 (8 h), and 325 °C (8 h) and subsequently post-cured under an inert atmosphere of nitrogen at 350 (8 h) and 375 °C (4 h) is shown in Figure 13. Saturated absorption conditions were reached after 31, 16, and 11 days for the neat polymer, phthalonitrile–EP0408 copolymer, and phthalonitrile–AM0281 copolymer, respectively. However, the amount of water absorbed at saturation did not follow this trend. Thus, the phthalonitrile–EP0408 copolymers showed the lowest saturation value among all the polymers tested (0.6%), whereas phthalonitrile–AM0281 showed the highest saturation value (1%), which was larger than that of the neat polymer (0.7%).

**Figure 14:**
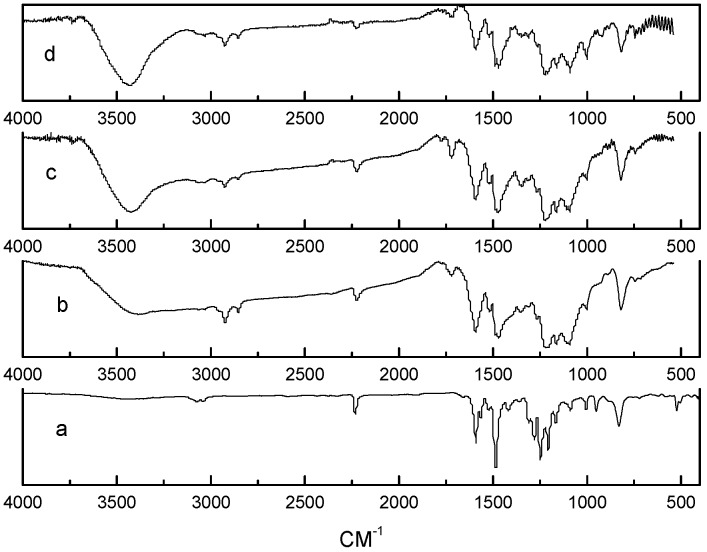
FTIR spectra of: (**a**) pre polymer, (**b**) neat polymer, (**c**) EP0408-0.5 polymer, and (**d**) AM0281-1 polymer.

**Figure 15:**
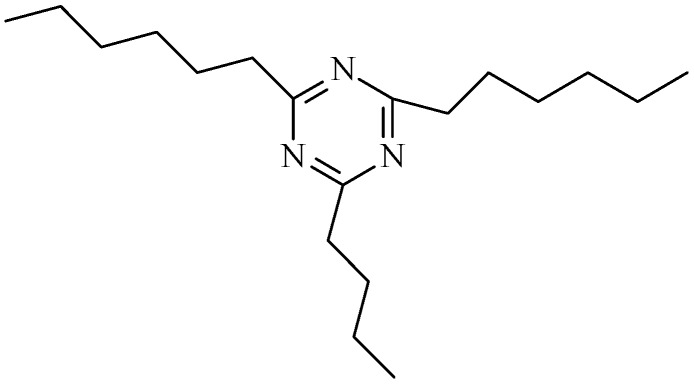
The structure of triazine.

Figure 14 shows the FTIR spectra of the neat prepolymer, the neat polymer, the EP0408-0.5 polymer, and the AM0281-1 polymer. FTIR was used to monitor the polymerization process. The evolution of the nitrile absorption peak (2234 cm^−1^) and the formation of new peaks were studied to obtain information on the polymerization mechanism (Figure 14). The nitrile band was more intense for the prepolymers compared to the cured polymers, confirming that the nitrile groups reacted at high temperatures. The weaker nitrile peak showed by the AM0281-1 sample revealed a more rapid polymerization for this material. The cured polymers showed noticeably different FTIR spectra compared to the prepolymers. The peaks centered at 1518, 1520, 1522 cm^−1^ and at 1352, 1368, and 1364 cm^−1^ (Figure 13, curves b–d respectively) were ascribed to triazine rings. Moreover, the carbonyl bands at 1713, 1725, and 1717 cm^−1^ (Figure 13, curves b–d respectively) were produced by the oxidation of the polymer at high temperatures. Based on these results, the potential structures of triazine and POSS within the triazine ring network were proposed and shown in Figure 7 and Figure 15 respectively. The POSS functionality is grafted to the polymer chains upon reaction with the matrix, occupying the triazine network (Figure 7). As a result, the cross-linked density of the polymer was increased, which led, in turn, to higher thermal stabilities.

## 4. Conclusions

The following conclusions can be drawn from this study:

Conclusion 1: The polymerization rates of neat and POSS-containing polymers were determined by the quantity and reactivity of the reactive groups in the curing system. The activation energies of for the neat polymers and the EP0408- and AM0281-containing polymers were 135, 129, and 78 kJ/mol respectively, indicating that the POSS-containing polymers had higher chemical reactivities and superior stability than the neat polymers. 

Conclusion 2: According to the DMA data (Figure 8), high curing temperatures and long curing times resulted in polymers with enhanced oxidative stabilities. 

Conclusion 3: According to the TGA measurements, the neat polymer showed higher weight losses at 900 °C (25% of initial weight remaining) than either the EP0408-0.5 (45% weight remaining) or AM0281-1 (40% remaining) polymers. Thus, polymers containing POSS showed superior thermal stabilities than the neat polymer.

Conclusion 4: As revealed by FTIR, the polymerization products were triazine ring structures, which are believed to be responsible for the good thermal properties of the modified polymers.
